# Book Reviews

**Published:** 1823-03

**Authors:** 


### CRITICAL ANALYSIS
### OF
### ENGLISH AND FOREIGN LITERATURE
### RELATIVE TO THE VARIOUS BRANCHES OF
### Medical Science.

Quæ laudanda forent, et quæ culpanda, vicissim

Illa, prius, cretâ; mox hæc, carbone, *notamus*.—PERSIUS.

### DIVISION I.

### ENGLISH.

ART. I.—*A Treatise on Dislocations, and on Fractures of the Joints.* By Sir ASTLEY COOPER, Bart. F.R.S. Surgeon to the King, &c. &c. &c.—1 vol. 4to. 30 plates, pp. 562. Longman and Co. London.

[Concluded from page 150.]

IN our last Number, we brought down our analysis of Sir Astley's work to the conclusion of the chapter on Compound Dislocations of the Ankle-joint; and we now resume the thread of our discourse.

*Fracture of the Fibula* is well known to be of frequent occurrence, and the signs of the accident are too familiar to render it necessary for us to enter much into detail. The snap described by our author to be felt at the moment, is not, however, an invariable criterion; and therefore we would advise the surgeon, in every severe strain of the joint, to examine carefully whether this bone be broken or not; for we have, upon one occasion, met with an instance where, in consequence of this neglect, the bone remained un-united, and proved a serious inconvenience to the patient in after-life. The mode of discovering this accident is "by rotating the foot with one hand, and by grasping the lower part of the leg with the other: at each rotation, a crepitus is generally felt." (p. 353.) The great object in the treatment of this fracture is to preserve the great toe in a line with the patella, and to place the leg on its side in a half-bent position: the application of a well-padded splint, with a foot-piece on each side, becomes therefore in this case indispensable; for, without it, the foot cannot be preserved in the proper position. From the neglect of this precaution, Dr. Blair, a naval physician, remained lame after an accident of this description; the foot becoming twisted, " so that he walked better upon an inclined plane than upon flat ground."

*Fractures of the Tibia* into the ankle-joint, or a little above it, are not difficult to be recognized, and their mode of treatment offers nothing novel. In both these cases, Sir Astley recommends the leg to be raised so as to bend and elevate the knee; the leg should rest upon the gastrocnemius muscle, and upon the heel. Our author prefers this position, because it gives the surgeon an opportunity of observing the least deviation in the line of the foot, and because it is easier to the patient.

" Oblique compound fractures into the ankle-joint generally do very well, if care be taken to procure adhesion of the wound, which is to be effected by applying lint embued in blood to the lacerated skin, and leaving it there until it separates spontaneously." The position must vary with that of the wound. Even if suppuration occurs, the patient will generally recover, unless he be much advanced in years.

This chapter, or division of the work, concludes thus:—" But if, with the compound fracture into the joint, there be much comminution of bone, and bleeding from any large vessel, it will be proper to amputate immediately,—more especially if the patient be obliged to obtain his bread by labour; for, after recovery from great comminution, the limb will bear but slight exertion."

*On Dislocation of the Tarsal Bones.*—The dislocation of the astragalus is the first in order; but it is a rare occurrence, though a most serious accident when it does take place; for the reduction is difficult, and a considerable degree of lameness ensues if it be not reduced. A case in illustration is added, in which, although extension had been employed to a great degree immediately after the accident, it was found impossible to restore the bone to its situation, and the appearance of the limb is as follows:—The toes are turned inwards, and pointed downwards; and there is but a slight degree of motion at the ankle. In these cases, the use of the pulleys will be required, and the action of the muscles should be lessened by doses of the tartarized antimony. Compound dislocation of the astragalus is, of course, still more serious in its nature; and there appears to be no alternative in these cases, but amputation of the limb or

1

removal of the bone; for the reduction, although mentioned to have been successful in one instance, is not otherwise hinted at in this chapter, which contains no further instructions than such as the reader will derive from the detail of some highly interesting cases of this accident; in four of which the bone was removed with the knife, and the patients recovered with tolerable motion of the joint. We shall detail the leading features of the two most interesting of the above cases; in one of which it will be seen that the attempts made to reduce the dislocation were unavailing. Sir Astley here very justly inculcates the propriety of surgeons being upon their guard in amputating limbs and performing operations, as the resources of nature are sufficient, under very formidable circumstances, to effect restoration; a sentiment in which we are most happy to coincide with him upon this occasion.

The first case we shall abridge is that of Mr. Downes, in which, although the dislocation was not exactly compound, "the bone was dislocated forwards and inwards, projecting so as to make the case perfectly clear, and bearing so strongly against the skin that a slight incision would have exposed it." The patient met with the accident on the 24th July, 1820, and had been visited by Mr. West, who had tried to reduce the bone into its situation, but which could not be effected. The patient was largely bled, the limb placed in splints, a goulard lotion applied, and an anodyne given. Sir Astley, who saw the patient on the following day, at first thought of dissecting away the astragalus, but finally determined to wait, in the expectation of the skin giving way; he therefore continued the previous treatment. On the following days, some local and general irritation of the system took place, which was combatted by leeches applied to the part, and the exhibition of saline antimonial medicines. On the 10th August, the skin became disposed to slough, opposite the projection at the inner ankle: fomentations and a yeast poultice were applied, and bark and wine administered internally. On the 16th, the skin sloughed; on the 20th, the astragalus became exposed. The same means were continued; the inflammation and discharge gradually lessened; the bone became gradually dislodged by the sloughing or ulcerating of the ligament. In September the patient was removed to London; and on the 5th of October, the astragalus being "very loose," Sir Astley removed it with the forceps, dividing only some slight ligamentous adhesions. Some exfoliations occurred in December, but at the end of that month he began to walk. In October 1821, Mr. Downes had slight motion of the ankle, which has been gradually recovering. This case is illustrated by a very beautiful plate.

The other case to which we have alluded above was under the care of Mr. Green, whose account of the nature of the accident we shall copy verbatim.

"Thomas Toms, twenty-three years of age, was admitted into St. Thomas's Hospital, on the 14th July, 1820. He had fallen, whilst engaged in his business, (that of a bricklayer,) from a three-story scaffold, and his descent had been arrested by his foot catching between the spikes of an iron railing, from which he hung with his head nearly touching the ground. A wound was found, extending beneath the inner malleolus of the left leg; and the head of the astragalus, which was torn from the articulatory surface of the os naviculare, protruded through the divided integuments. Part of the articulatory cartilage of the displaced bone had been separated, and the bone was girt by the edges of the wounded skin, which was puckered under it. The tendons of the tibialis anticus and of the flexor muscles were tightly stretched, and the foot was turned rather upwards and outwards. Further examination showed that the posterior tibial artery was torn through, and that the accompanying nerve was partially lacerated. An attempt was made to reduce the luxated astragalus, by fixing the knee, after having bent the leg upon the thigh, and by making extension of the foot directly from the leg, laying hold of the heel with one hand, and placing the other on the dorsum of the foot. This however failed; and, as it appeared that the skin which firmly embraced the bone beneath prevented the replacement, it was divided, and the extension renewed, but with the same unsuccessful result. This difficulty seemed to arise from the small size of the wound in the capsule of the joints, and in consequence of the bone being tightly held by the tendons." (p. 371.)

Under these circumstances, Mr. Green was induced to consider whether amputation ought not to be proposed; but Sir Astley Cooper, after carefully examining the limb, suggested that the astragalus might be removed. This was accordingly done by Mr. Green, who first tied the posterior tibial artery, and then the bone was dissected from the ligaments without much difficulty. The parts were then brought together, and the wound closed with adhesive plaster; the leg was placed on its outside on a well-padded splint, with a foot-piece; the foot was supported above the level of the knee; and an evaporating lotion constantly used. We do not think it necessary to detail the symptoms that ensued from day to day: constitutional irritation, swelling, inflammation, and suppuration, took place, but not to any alarming degree. On the eighth day, it appears that the pulse was 86, and the wound had a healthy granulating appearance. He continued to recover from this time until the 29th of July, when an abscess formed; but which was opened on the 1st of August, and the feverish symptoms disappeared. Two other abscesses, and a sinus running up the calf of the leg, afterwards contributed to retard his recovery; but, by the 25th of October, the discharge had entirely ceased, and he was capable of performing flexion of the foot to a considerable degree, but could not extend it. He was discharged from the hospital on the 2d November, and has since been enabled to resume his usual occupation.

We pass over a few pages, and pause at the chapter on *Dislocation of the Jaw*, in order to relate the following method of reducing it, which in many instances possesses great advantages over the method commonly practised. A madman dislocated his jaw, and Sir Astley very properly adds:

"I thought a surgeon must be as insane as the patient who would employ the usual means of reduction, and I therefore desired the keepers to place the patient on a table upon his back, with a pillow under the head, and that he should be held by several persons. I ordered two table-forks to be brought me, and wrapped a handkerchief round their points; then, placing myself behind the patient's head, I carried the handles of the forks into the mouth, on each side, behind the molares teeth: then directed them to be held; and then, placing my hand under the chin, I forcibly drew it to the upper jaw, which was used as a lever upon them, depressing the processes as the jaw was elevated, and thus directing the bone backwards into its natural situation. But, as wood is liable to injure the gums, it is better to substitute two corks, which are to be placed behind the molares teeth, on each side of the mouth, and over these the chin is to be raised." (p. 390-1.)

A piece of wood has sometimes been used as a lever, first on one side, and then on the other; but our experienced author prefers the corks, the recumbent posture, and the elevation of the chin. Great relaxation of the ligament of the jaw sometimes occurs, especially in young females; and a *snapping* is felt in the maxillary articulation, arising from the jaw suddenly slipping into its socket, which the relaxation had permitted it to quit. The internal exhibition of steel and ammonia, the shower-bath, and a blister applied before the ear, have been found usually to remove this troublesome complaint. In a case of this kind which occurred to us some years back, electricity alone removed the complaint.

We pass by the account of *Dislocations of the Clavicle*, as containing nothing remarkably novel: they are not of very frequent occurrence, nor are they difficult to detect or to reduce; but it must be remembered, says our author, that, after the utmost care in the treatment, "some slight deformity will remain; and it is necessary, from the first moment of the treatment, that this should be stated to the patient, as he may otherwise suspect that it has arisen from the surgeon's ignorance or not." (p. 408.)

The chapter on Dislocations of the Os Humeri is preceded by a concise, but very perspicuous, description of the structure of that joint, but which we do not deem it necessary to detail.

*Dislocation of the os humeri* may take place in four directions: three of these are complete, and one is partial. The first is downwards and inwards, or into the axilla. The second is forwards under the pectoral muscle, when the head of the bone is placed below the middle of the clavicle, and on the sternal side of the coracoid process. The third is backwards, when the head of the bone can be both felt and distinctly seen, forming a protuberance on the back and outer part of the inferior costa of the scapula, and is situated upon its dorsum. The fourth is only partial, when the anterior portion of the capsular ligament is torn through, and the head of the bone is found resting against the coracoid process of the scapula, on its *outer* side.

Sir Astley has never seen the os humeri dislocated upwards: it can only happen in conjunction with fracture of the acromion.

On the comparative frequency of these accidents, our author says that he has seen a *multitude* of instances of the dislocation into the axilla; of the forward one, *several*; and of the dislocation backwards, only *two*, in a practice of thirty-eight years. He does not believe that, after dislocation, any change takes place, the muscles having once contracted; so that, excepting from circumstances of great violence, the nature and direction of the dislocation are not subsequently altered.

The usual signs of the dislocation into the axilla are too well known to need repetition here; but Sir Astley observes, that the head of the bone cannot be felt in the axilla unless the arm be removed from the side; and, from a neglect of which precaution, he has seen surgeons deceived, and give a wrong opinion as to the nature of the accident. It must be recollected that, though certain motions of the limb are lost in consequence of this dislocation, much difference in the degree of that motion will arise in the age of the patient; for, in old people, the relaxed state of the muscles will sometimes permit the arm to be carried to the upper part of the head. Crepitus sometimes is slightly felt on first moving the limb, but by a continuance of the motion it ceases. This accident is therefore principally detected by the fall of the shoulder, the presence of the head of the bone in the axilla, and the loss of the natural motions of the joint; but, as these circumstances become obscured after a few hours, by extravasation of blood and the great swelling that ensues, and which after a lapse of some time becomes absorbed. If, says our author, "we at this period detect a dislocation which has been overlooked, it is our duty, in candour, to state to the patient, that the difficulty in the detection of the nature of the accident is exceedingly diminished by the cessation of inflammation and the absence of tumefaction." (p. 419.) This is liberal and candid! Would to heaven that we met with it as often in practice as in precept.

An account of the dissection of two cases of dislocated shoulder is subjoined, which afford the following important considerations. After having exposed and described the lesions of the muscles and the condition of the joint, our author next endeavoured to reduce the bone, but found the resistance such as he could not overcome, and he therefore proceeded, by dividing one muscle after the other, to ascertain from whence the resistance proceeded: he found the supra spinatus muscle to be the great opponent; but, when this was relaxed by raising the arm directly upwards, the head of the bone glided into the glenoid cavity. This leads to the practical conclusion, that, in this dislocation, the arm should be raised horizontally, rather than brought obliquely downwards, and the biceps is to be relaxed by slightly bending the arm. In an old dislocation, which had been long unreduced, the following circumstances were observed:—"The head of the bone is altered in form, the surface towards the scapula being flattened, and a complete capsular ligament covers the head of the os humeri. The glenoid cavity is completely filled by ligamentous matter; small portions of bone are suspended in this ligamentous matter, which appear to be of new formation, as no portion of the scapula or humerus is broken. A new cavity is formed for the head of the os humeri, on the inferior costa of the scapula; but this is glenoid, as that from which the os humeri had escaped." The shoulder, when once dislocated, is known to be frequently very liable to a recurrence of the accident, the slightest motion of the arm sometimes causing the displacement of the bone,—of which two or three instances are mentioned: but this may be prevented by fixing the arm to the side for two or three weeks, during which time the ruptured tendons and ligament will become united, an event which the motion of the arm either impedes or wholly prevents.

*Of the Reduction of the Dislocation in the Axilla.*—Many methods of replacing this dislocation have been recommended; that which Sir Astley usually adopts in his private practice is performed as follows:—The patient is placed in the recumbent position; the surgeon then binds a wetted roller round the arm just above the elbow, upon which he ties a handkerchief; then, with one foot resting upon the floor, he separates the patient's elbow from his side, and places the heel of his other foot in the axilla, receiving the head of the os humeri upon it; he then draws the arm, by means of the handkerchief, *steadily* for three or four minutes, and, under common circumstances, the head of the bone is easily replaced. If more force should be required, a long towel may be substituted for the handkerchief, by which several persons may pull. The fore-arm should be bent at right angles with the arm. Extension may be made from the wrist, but it requires more force, although it has this advantage—the bandage is not liable to slip. Should the above plan fail,

(which, if the person be very muscular, or the accident has happened some time, it frequently will,) then more force is required. We need not describe the minute steps of a process so universally known, but we cannot too often repeat the very important rule, that to fix the scapula is the *principal* object before extension of the arm be employed. The bandage used at Guy's Hospital for this purpose is a girt buckled on the top of the acromion, so as to raise the bandage nigh in the axilla. The extension is to be made slowly and steadily, the arm being either at right angles with the body, or rather above it. Sometimes a gentle rotatory motion of the arm during the extension diminishes the opposition of the muscles.

The third method, of reduction by pulleys, is resorted to when both the above methods fail, and is applicable principally to those dislocations which have remained a considerable time unreduced. Sir Astley observes, that the pulleys are not employed simply in order to obtain a greater degree of power, but on account of the gradual and equal manner in which the force can be employed. There is no difference in the position of the patient, nor in the bandages employed in this latter case; but the surgeon should first draw the pulley himself, and, when pain is complained of, he should cease to draw, keeping up the degree of extension, and conversing with the patient, to direct his mind to other objects. After a pause of two or three minutes, the extension may be renewed until pain is again complained of; thus alternating for a quarter of an hour, at intervals rotating the arm. The bone, in this instance, generally passes into the socket without the snap which is heard when other means are employed. Sir Astley, in his hospital practice, employs bleeding, the warm bath, and nauseating doses of tartarized antimony, previously to employing mechanical means; and these very much lessen the necessity of employing very considerable force. Mr. H. Cline was in the habit of directing his patients to support a weight for some time before extension was begun, in order to fatigue the muscles. A small cushion in the axilla, and the stellate bandage, together with a sling to support the arm, should be worn for some time after. The simple method of placing the knee in the axilla, first separating the arm from the side, and then pulling the arm downwards with one hand, whilst the other rests upon the acromion scapulæ, has frequently succeeded, says our author, even with muscular persons, when in a state of intoxication. The plan recommended by Mr. Kirby, of Dublin, is then described, and spoken of with approbation.

The *Dislocation forwards*, or behind the pectoral muscle, is more distinctly marked than that just described; as the hollow below the acromion is much more considerable, and the head of the bone can be distinctly felt, and in thin persons even seen, and which rotation of the arm makes more evident. The arm is also somewhat shortened, and the elbow is thrown more from the side than in the former case. We shall omit noticing the dissection of this accident, because it does not lead to any practical inference, and proceed at once to the mode of reduction; and upon this point we have merely to observe, that the bone must be drawn downwards and a little backwards; and, if the foot is placed in the axilla, it is requisite to bring it more forward. In those cases where it is necessary to use pulleys, the extension must be kept up longer than in the former dislocation, as the resistance is greater.

*Dislocation of the Os Humeri on the Dorsum Scapulæ.*—Only two cases of this accident have occurred at Guy's Hospital in thirty-eight years. It cannot be mistaken, as the head of the bone forms a protuberance upon the scapula, which, when the elbow is rotated, moves also; the dislocated bone may also be easily grasped between the fingers, and distinctly felt resting below the spine of the scapula. The first case which occurred was reduced in a few minutes by the same means as those employed in the dislocation in the axilla; and the second case was treated, with equal success, in a similar manner. Three other cases of this kind are recorded; one by Mr. Toulmin, and two by Mr. Coley, of Bridgnorth.

*The partial dislocation of the os humeri* is not a very uncommon accident, and the following are its usual marks:—There is a depression opposite the back of the shoulder-joint, and the posterior half of the glenoid cavity is perceptible, from the advance of the head of the bone; the axis of the arm is thrown inwards and forwards; the elevation of the arm is prevented by the head of the humerus striking against the carocoid process; and there is an evident protuberance formed by the head of the bone in its new situation, which is felt readily to roll when the arm is rotated; in this accident the anterior part of the capsular ligament is torn. The mode of reduction is the same as that employed in the dislocation forwards; but it is necessary to draw the shoulders backwards, to bring the head of the bone to the glenoid cavity; and, as soon as the reduction is effected, the shoulders should be bound back by a clavicle bandage, or the bone will immediately slip forwards again. Our author observes that, where dislocation is complicated with fracture of the head of the os humeri, reduction is much easier than in simple dislocation, as the insertion of the principal opponent muscles, the supra and infra spinati, is removed; but it renders it more difficult to retain the bone within the glenoid cavity, when it is replaced.

A case of compound dislocation of the shoulder-joint forwards, which occurred in the practice of Messrs. Saumarez and Dixon, of Newington, is narrated. The patient, aged fifty-five, was intoxicated at the time the accident happened. There was no difficulty in returning the os humeri into its situation; but, of course, great constitutional disturbance ensued, and the wound suppurated copiously; abscesses formed in various parts round the joints, and sinuses resulted from some of them. Nevertheless, at the end of fourteen months, the man recovered with an anchylosed joint, but with free motion of the fore-arm, which enabled him to handle his pen for all the purposes of business.

It has occurred to the writer of this article to see the head of the os humeri shattered by shot and splinters of shells upon several occasions. Two of these cases, in which amputation had not been performed in the first instance, terminated favourably,—the whole head of the bone being discharged from the wound, in three or four successive portions. Abscesses, of course, were formed in the neighbourhood of the joint, and the constitution sympathised with the local injury, but not, at any moment, to such an extent as to make the ultimate recovery of the patient at all doubtful. The joint was anchylosed, but the lower arm was capable of performing its usual offices.

From this digression we return to notice a few accidents which are liable to be confounded with dislocation of the shoulder-joint. The first is *fracture of the acromion*. On this occasion the roundness of the shoulder is lost, part of the attachment of the deltoid muscle being broken off; the arm sinks towards the axilla as far as the capsular ligament will permit; but, upon raising the arm, the form of the shoulder is at once restored; and, on tracing the acromion from the spine of the scapula to the clavicle, a depression is felt at their junction. The best way, therefore, to detect this accident is to raise the elbow, and then rotate the arm, when a crepitus will be felt at the point of the shoulder. The patient, directly after the accident has happened, feels as if the arm was dropping off, with a great sense of weight, and but little power to raise it. Bony union will take place in this fracture if the parts can be kept in contact. The best method to ensure this is to raise the elbow, and to fix the arm; and, if it be kept steadily in that position, it will support the broken process, and keep it in its place: a cushion should be placed in the axilla, to relax the deltoid muscle; the arm should then be bound to the chest by a roller, and kept in that situation for three weeks.

*Fracture of the neck of the scapula* is illustrated by the case of a young lady who was thrown from a gig, and who was told by the surgeon who was first sent for that her shoulder was dislocated: by extension, all appearance of dislocation was removed, and the arm was bound up; but the next day the arm reassumed all the marks of dislocation. Sir Astley, who was requested by the surgeon to see the patient, by raising the arm from the elbow, and the head of the bone in the axilla, restored the natural appearance of the parts, but, as soon as the support was removed, the arm immediately sunk: he then rotated the elbow, and pressing the coracoid process of the scapula with his fingers, distinctly heard a crepitus. A thick cushion was placed in the axilla, and the bone was retained in its situation by a clavicle bandage for seven weeks, when it became united without deformity. Our author remarks, that the laceration of a ligament which passes from the under part of the spine of the scapula to the glenoid cavity, and which is not usually noticed in anatomical works, is the cause of the deformity in this case.

*Fracture of the neck of the os humeri* sometimes occurs in the young and in the old, seldom in middle age. In this case the body of the humerus sinks into the axilla, and the roundness of the shoulder is lessened; the arm cannot be supported, nor the elbow raised from the side, without the assistance of the other hand; crepitus could not be felt by rotating the arm, but by raising the bone and pushing it outwards. The treatment consists in rolling the arm from the elbow to the shoulder, applying a splint both on the inner and outer side, placing a cushion in the axilla, and supporting the arm gently with a sling. If the arm be too much raised, the bones may overlap, and deformity will ensue.

The great length to which this article has already extended compels us to be concise in our notice of the remaining portion.

Of *dislocations of the Elbow-joint* five different species are enumerated:—1st, the dislocation backwards of both bones; 2dly, their displacement laterally; 3dly, the dislocation of the ulna separately; 4thly, the dislocation of the radius forwards; and, 5thly, the dislocation of the same bone backwards. The first accident is strongly marked, and cannot well be mistaken. The mode of reducing it is, either by placing the patient in a chair, when the surgeon applies his knee to the inner side of the joint in the bend of the arm, and, taking hold of the patient's wrist, he bends the arm, at the same time pressing on the radius and ulna with the knee: or the patient's arm may be placed round the post of a bed, and forcibly bent in this situation. This dislocation may be reduced even after the lapse of many weeks. When the bones are replaced, the arm should be bandaged in the bent position, and put in a sling.

The second, or lateral dislocation, may be reduced by the same means, and with the same facility as the former; and it is also well marked.

The dislocation of the ulna backwards produces much deformity of the limb, the fore-arm and hand being twisted inwards.

It is rather difficult to detect, but it is more easily reduced than when both bones are displaced. The best method is to bend the arm over the knee, and to draw the fore-arm downwards.

Of the dislocation of the radius forwards, Sir Astley has seen six examples; the marks are the following:—The fore-arm is slightly bent, but cannot either be perfectly extended or brought to a right angle; when it is suddenly bent, the head of the radius strikes against the fore-part of the os humeri, and neither pronation nor supination can be perfectly performed; and, if rotation of the hand be attempted, the bone will be seen to roll. In this case the head of the bone rests in the hollow above the external condyle of the os humeri. It appears to be an accident difficult to reduce. In the two first cases mentioned by our author, all attempts were unsuccessful; in the third and fourth cases, the bone was replaced. The last patient was placed upon a sofa, and the arm was bent over the back of it, and then extension was made from the hand, without including the ulna; and this seems to be the most likely mode of succeeding.

Sir Astley has never met with an instance of the dislocation of the radius backwards in the living subject.

Fracture of the condyles of the os humeri just above the joint, may be easily mistaken for the backward dislocation of the radius and ulna. The distinguishing marks are, that, by rotating the arm, a crepitus may sometimes be felt; and the appearances are removed by extension, but return the moment the extension ceases. It is an accident more liable to occur to children than adults. It is to be treated by bending the arm and drawing it forwards, and then rolling it up; the best splint is one bent at right angles, placed behind the upper arm, the lower portion under the fore-arm. The arm must be kept in the bent position. If the patient be young, passive motion may be begun in a fortnight; but, under the best treatment, there is, says our author, sometimes considerable loss of motion.

Sir Astley mentions a fracture of the coronoid process of the ulna; of which one case, which was unreduced, is mentioned. He seems to doubt whether this accident can be rectified; "as the coronoid process, like the head of the thigh-bone, loses its ossific nourishment, and has no other than a ligamentous support." (p. 485.) It is however proper to keep the arm steadily bent for three weeks after the injury, that the ligamentous union may be as short as possible.

We shall pass over the fracture of the Olecranon, as both the nature of the accident and the mode of treating it are pretty universally understood; and the nature of the accident is so clear as to render it scarcely possible to confound it with any other.

We also pass over many pages descriptive of a variety of minor accidents, all highly necessary to be known by the surgeon, but which our limits will not permit us to dwell upon, in order to mention *dislocations of the Thumb*, because they are accidents very difficult to reduce, on account of the numerous strong muscles which are inserted into this part. In the dislocation of the metacarpal bone from the os trapezium, the bone is usually thrown inwards; the thumb is bent backwards, and cannot be brought towards the little finger. As the flexor muscles are so much stronger than the extensors, it is best to incline the thumb towards the palm of the hand during extension, which must be steadily kept up for a considerable length of time: but if simple extension, carried to the extent that prudence warrants, does not succeed, it is best to leave the case to nature. Of compound dislocation, one very interesting case is mentioned, in which that accident occurred from an explosion of gunpowder. The surgeon, Mr. G. Cooper, of Brentford, who first saw the patient, perceiving that both the flexor and extensor tendons were uninjured, restored the bone to its situation; and the result was recovery, with very useful motion of the thumb. We need not detail each separate form of these dislocations, but merely observe, that the plan of reduction is essentially the same in all: the object is to relax the flexor muscles as much as possible; the hand should then be steeped in warm water, to contribute towards the relaxation of the parts; a piece of wetted leather is next to be closely applied round the first phalanx of the thumb; a portion of tape, about two yards in length, is then placed upon the leather, in that form of knot called by sailors the "clove hitch," and which is drawn tighter as the extension proceeds. "An assistant places his middle and fore-finger between the fore-finger and thumb of the patient, and makes the counter-extension; whilst the surgeon, assisted by others, draws the first phalanx from the metacarpal bone, directing it a little inwards towards the palm of the hand." (p. 533.) If this plan does not succeed, we are directed, after having applied the leather and sailor's knot as above, to place a strong worsted tape between the metacarpal bone of the thumb and fore-finger; the arm is then to be bent around a bed-post, and the worsted tape fixed to it; a pulley is hooked to the tape which surrounds the first phalanx, and extension is then to be made. This method, it is added, is almost sure to succeed. In compound dislocations of the first or second phalanx, our author thinks it best, if reduction cannot be effected, to saw off the extremity of the phalynx.

A few observations on dislocations of the Ribs brings us to the last division, on *Injuries of the Spine.* It has been generally stated by surgeons, that dislocations of the spinal column frequently occur; but, if luxation, says Sir Astley, *ever does*

*happen*, it is an injury which is "extremely rare, as, in the numerous instances which I have seen of violence done to the spine, *I have never witnessed* a separation of one vertebra from another, through the intervertebral substance, without fracture of the articular processes; or, if those processes remain unbroken, without a fracture through the bodies of the vertebræ." (p. 539.) We conceive a few simple experiments upon the dead body might set this question at rest; for, as our neighbours, not long ago, tumbled dead bodies down stairs, and threw them from considerable heights, to ascertain the possibility of rupturing the liver by the concussion, the same plan might perhaps be applied more rationally in order to ascertain whether external injuries can dislocate the spinal column, exclusive of fracture. Of course, these observations apply to the dorsal vertebræ principally; it is well known that the upper cervical are occasionally dislocated. Sir Astley gives us a clear and excellent anatomical description of the vertebral column; but we must draw to a conclusion, observing that, in the fractures and displacements of this column, whatever plan be adopted, the result is uniformly unfortunate. The case in which Mr. H. Cline trepanned the fractured vertebræ, with the intention of relieving the spinal marrow from pressure, is recorded. The case was unfortunate; and it is added, that Mr. H. Cline was blamed for making the trial. We have already, in another place, stated our reasons for believing that this operation is not likely to prove successful; but we are far from attaching blame to the attempt: on the contrary, we look upon it as rational and scientific, and highly deserving of our warmest approbation. It is only by such means that our doubts can be resolved, and our art brought to perfection.

We have now finished our analysis of this long and interesting work: we have dwelt chiefly upon those parts which we thought the most important, and the least generally understood. The space which we have devoted to our task is a sufficient proof of the importance we attach to it; and we shall make no apologies to Sir Astley for the few criticisms which we have hazarded upon particular points: they are put forth in a plain, honest, and (we hope) a respectful manner; and we are much mistaken if the eminent author himself (even should he be disposed to deny the correctness of our deductions,) will not be the foremost to admit that they have been urged with a due attention to those rules of conduct which we promised not to lose sight of when we first engaged in the dangerous profession of criticism.*

* We have to acknowledge the receipt of a polite letter from Mr. Eves, of Colford, pointing out a mistake in the former part of this analysis. In mentioning the dislocation of the femur downwards, we have said, (p. 137,) "the limb is *shorter* than the other:" the word ought obviously to have been *longer*.—Editors.

Art. II.—*Historical Sketch of the Opinions entertained by Medical Men respecting the Varieties and secondary Occurrence of Small-Pox; with Observations on the Nature and Extent of the Security afforded by Vaccination against Attacks of that Disease.* In a Letter to Sir James M'Grigor, Director-General of the Army Medical Department. By John Thomson, M.D. F.R.S.E. &c. &c. &c.—8vo. pp. 400; Appendix, 43. Longman and Co. 1822.

All who are interested in the discussions which have of late years taken place with regard to small-pox and vaccination, must feel indebted to the author of the present work. Indeed, the patient observation with which he has watched the phenomena of varioloid diseases, and the laborious researches he has made into the records of medicine, tracing the history of small-pox epidemics, have been such as to render his name most honourably associated with this, probably, the greatest medical question which can occupy the attention of the physician or political economist. It is not our intention, however, to eulogize Dr. Thomson, but give our readers as distinct an analysis of the work before us as the limited space we can devote to it will admit. In a former work,* Dr. Thomson devoted much ingenious argument and illustration to show that all varioloid eruptions are specifically the same,—arising from the operation of one contagion, and varying in form with varieties of constitution and other incidental circumstances. This question, however, was so fully and so ably discussed by our ingenious friend and predecessor, Dr. Hutchinson, that we deem it unnecessary to go over the ground a second time.

Dr. Thomson having found, during the late epidemy, that small-pox frequently occurred in persons who formerly had passed through that disease, became desirous to ascertain how this fact stood with regard to small-pox epidemics in general, as their histories are given by the older writers, as well as by those of more recent date. With this view he entered upon a course of laborious inquiry, the results of which form the object of the present work.

Dr. Thomson, for the purpose of simplifying this investigation, has arranged his observations under three great divisions. The first of these contains the history of small-pox prior to the discovery of inoculation, embracing an epoch of nearly eight centuries; the second, its history from the introduction of small-pox inoculation at the beginning of the eighteenth century, to the discovery of vaccination, a period of about eighty years; and, lastly, from the introduction of cow-pox up to the present day. One of the principal questions to be determined is this—Has small-pox been generally believed, by practical au-

* *An Account of the Varioloid Epidemic in Edinburgh and other parts of Scotland.*

thors, to occur only once during life; or may it attack the same individual a second or third time? Some authors answer in the affirmative; others, on the contrary, particularly the later writers, deny the recurrence of this disease. At first sight, it must seem remarkable that a question, apparently simple, should have been so long involved in doubt. This appears to have arisen, in a great measure, from the great diversities of form assumed by the disease, and, during the last century, from the summary process of reasoning which became current on the subject: for, it having been judged expedient, by the supporters of small-pox inoculation, that their patients should not have the disease a second time, if they were unfortunately attacked with a suspicious eruption, it was argued thus,—*because* the former disease communicated by inoculation was small-pox, *therefore* this is something else; a species of sophistry which has since been too frequently had recourse to by the *ultra* vaccinists of our own times.

The causes on which the varieties in the appearance of small-pox, above alluded to, have been supposed to depend, are—differences of constitution, states of atmosphere, situation and treatment, the communication of the disease, whether natural or artificial; and, lastly, differences in the nature of the contagion. "Great, if not insurmountable difficulties," says our author, "unquestionably arise in tracing the diversities of small-pox, which occur in individual instances, to the particular or combined operation of these different causes; and there seems but too much reason to believe that medical writers, in accounting for the diversities of this disease, have not always attended sufficiently to those difficulties, but have often referred diversities to the operation of one of those causes, which were, in fact, attributable to the separate or combined operation of others."

*First Period.*—In his account of the first period of small-pox, Dr. Thomson has referred to the opinions of more than fifty writers; from these we shall make a few selections. RHAZES describes the effects of individual constitution in modifying the appearances of the disease, and gives such an account of its different varieties as show him to have been familiar with them. He has pointed out the distinguishing characters of the distinct, coherent, and confluent kinds,—the regular and anomalous,—the mild and the malignant; but he did not regard the milder form as securing the system from a recurrence of the disease; and asserts, on the contrary, that the same person sometimes suffers a second, or even a third, attack. That small-pox had attracted much attention at a very early period, may be inferred from the minuteness with which some of its varieties appear to have been described; two of the most singular of which, observed by Dr. Thomson during the late epidemy, were noticed by the Arabians. In the former, one pock, or more, is included in another; in the latter, fresh pox are formed, in the progress of the eruption, on the tops of those which first come out. AVICENNA, who mentions this variety, says that the same person frequently has small-pox twice. Indeed, of the Arabians, AVERRHOES is the only one who maintains an opposite opinion.

DUNCAN LIDDLE, of Aberdeen, who was professor of medicine at Helmstadt, towards the end of the sixteenth century, says that almost all men have small-pox once during their lives, a few twice, and very few a third time. MAYERNE mentions a case in which the small-pox occurred before the scabs of the first eruption had dropt off; and BOREL, in his "Historiæ et Observationes rariores," relates an instance of a woman, who, having passed safely through the small-pox seven times, died of the eighth attack in her 118th year!

MORTON published his Treatise on Small-Pox in 1694, in which he has mentioned various forms of the disease; and in particular alludes to a mild vesicular eruption, which, he says, runs its course without secondary fever, and is called *chicken-pox*. Long before Morton's time, medical writers had described many varieties of eruption under a corresponding variety of designations: among the most remarkable of these is a vesicular disease mentioned by VIDUS VIDIUS, under the name of *crystalli*, which he considers as essentially distinct from small-pox; but Dr. Thomson thinks it difficult to determine whether he meant to designate lymphatic small-pox, herpes, or pemphigus. "This employment," says Dr. T. "of the word *chicken-pox* (by Morton) is generally understood, I believe, to be the first time in which this term is used by any medical writer in England." Two years after Morton's treatise, an account of small-pox and measles was published by GIDEON HARVEY, in which reference is made to *swine-pox*. "They (the small-pox) appear, says Harvey, either in a greater number or lesser, of which latter the bigger are called by the doctrices the *swine-pox*, and the lesser the *chicken-pox*."

Without following Dr. Thomson through his review of the opinions of the older writers any farther, we shall lay before our readers the conclusions at which he arrives. These are, that a distinction was very early made of small-pox into proper and improper, genuine and spurious; that all the varieties of this disease have generally, if not universally, been supposed to have had one common origin; and, lastly, that many examples of its recurrence are mentioned by writers before the commencement of the eighteenth century.

*Second Period.*—Although the opposition was great to the introduction of small-pox inoculation in England, yet it seems to have been known from very remote antiquity among some other nations. In Mr. MOORE's History of Small-Pox, mention is made of various operations more or less similar to that employed in this country. The most ancient is that said to have been used by the Chinese, under the figurative name of *sowing* the small-pox.* They took a few dried small-pox crusts, as if they were seeds, and planted them in the nose; a bit of musk was added, in order to correct the virulence of the poison, and perhaps to perfume the crusts, which were always taken from a healthy person who had had the small-pox favourably. Some physicians had the crusts beat into powder, advising its use in the form of snuff. The Bramins are said to have communicated the disease by making scratches in the skin, and applying a piece of cotton soaked in variolous matter. The operation varied in different countries, but always consisted in puncturing or scratching the skin, by which means the matter was introduced.

The first persons inoculated for the small-pox in this country were the daughter of the elegant and accomplished Lady Mary Wortley Montague, and a child of Dr. Keith's, one of the physicians who had witnessed the former case. Soon after followed six condemned felons, and the two daughters of Caroline Princess of Wales: the felons found the operation a pleasant substitute for the gallows, and the young princesses passed happily through the disease. Notwithstanding these favourable results, the inoculation of small-pox advanced very slowly, and met with opposition at every step. Dr. Wagstaffe went so far as to say, in alluding to the eruption produced by inoculation, that "there was nothing like the small-pox, either in symptoms, appearances, advancing of the pustules, or course of the distemper." In 1755, Turgot caused a child to be inoculated; and, the following year, the Duke of Orleans had his son and daughter inoculated with success. M. Turgot and several of the nobility then submitted to the operation, which, having thus been rendered fashionable, was soon more widely practised.

We have already hinted at the injudicious length to which the friends of inoculation carried their assertions in its favour; for it has been shown by Dr. Thomson, in the most satisfactory manner, that, prior to this period, the great majority of medical writers acknowledged the possibility of small-pox recurring: whereas, now it was argued, that passing through the disease from inoculation gave greater security against a relapse than having it in the natural way. "If it were proved," says Condamine, "that small-pox may return a second time *naturally*, it does not follow that this will happen after inoculation." Suspicious eruptions, however, did occur; and it is amusing to observe the manner in which the difficulty was attempted to be got over.

* Moore's History of Small-Pox, p. 219, &c.

From this time the possibility of small-pox affecting the same individual more than once, became the subject of keen discussion, in which many of the most eminent medical men in Europe took a part. The uncertainty of the data on which their opinions rested, appears from the great discrepancy in the calculations with respect to the number of individuals affected with secondary small-pox. According to TISSOT, these are as one to 100; according to HEBERDEN, as one to 5000; and, according to CONDAMINE, as one to 10,000. It would have been well if, instead of injuring the reputation of inoculation, by claiming for it powers of prevention not verified by experience, medical men had followed the judicious admonition of M. LECAT, in a letter upon this subject. "Let us be contented," says he, "with these precious advantages of inoculation: they are the only solid principles of its success, and of its great superiority over the natural small-pox, demonstrated by the most universal and exact calculation. To push our pretensions further, is to indulge in chimeras and the wonderful,—it is to imitate the enthusiasts for novelty,—it is to lend weapons to the enemies of inoculation. Nothing is more pernicious to true religion than superstition and false miracles."

According to Dr. Thomson, the medical practitioners of Holland seem at all times to have believed in the possibility of a recurrence of small-pox, and their writings contain a great number of well-authenticated cases of this kind. Among others, the celebrated CAMPER, in a work published in 1774, states that, notwithstanding the possibility of this disease returning a second time was denied in England, he had been led to a contrary opinion, both from the credit due to those who had recorded cases of this kind, and also from the numerous examples which had fallen under his own observation.

On reviewing the opinions entertained by medical men with regard to small-pox, from the period of the introduction of inoculation, Dr. Thomson is led to conclusions, which we subjoin in his own words.

"1st. That most, if not all, of the advocates for this practice denied the possibility of the secondary occurrence of small-pox, and were anxious to disprove this opinion, conceiving that its admission might be injurious to the cause of inoculation.

"2dly. That, notwithstanding this unwillingness on the part of inoculators to admit the occurrence of secondary small-pox, cases of this kind have often presented themselves to the observation of medical practitioners, in the epidemic prevalence of this disease at different times, and in various countries of Europe; and that these cases have occurred in circumstances, and with symptoms, which do not appear to have left any room for doubt with regard to the genuine nature of the disease.

"3dly. That the varioloid eruptions occurring in those who had previously passed through either natural or inoculated small-pox, have had the appellations applied to them which had been given during the first period to spurious or illegitimate small-pox,—such as wind, water, horn, sheep, swine, and stone-pox, but apparently without the belief that these diseases arise from more than one contagion.

"4thly. That the opinion suggested by Dr. Fuller, that all the varieties of spurious small-pox are specifically different from one another, as well as from the genuine small-pox, does not appear ever to have been adopted by medical practitioners.

"5thly. That the crystalline, or lymphatic varioloid eruption, was considered by some of the advocates for inoculation in France as an eruption different, not only from small-pox, but also from chicken-pox.

"6thly. That all the different forms of the varioloid eruptions which had been previously considered by medical practitioners as spurious small-pox, were, about the year 1767, conceived by Dr. Heberden to arise from a contagion specifically different from that of small-pox; and that to all the diversities of these eruptions he gave the specific appellation of the varicella, or chicken-pox, and, by means of this hypothesis, was enabled to account for the apparent secondary occurrence of small-pox.

"7thly. That, though this opinion of Dr. Heberden was almost universally adopted by medical practitioners, yet that a few practical observers were, on particular occasions, under the necessity of regarding that form of varioloid eruption, which had usually been denominated swine-pox, to be specifically different from chicken-pox, as well as from small-pox, because they had observed it to occur in those who had previously passed through both of these diseases.

"8thly. That chicken-pox was declared to be a disease equally capable of being communicated by inoculation as small-pox, and that many examples have been recorded, by the first medical authorities, of the inoculation of chicken-pox.

"9thly. That, notwithstanding the prevalence of the opinion of the distinct nature of chicken-pox and of small-pox, and the assertion, so frequently repeated, that these two diseases had been observed to prevail separately, there is no satisfactory proof to be found in our medical records of their ever having done so; but many proofs of the fact, that all the varieties of the genuine, as well as of the spurious sorts of small-pox, have in the same epidemics come in and gone out together, in the same manner as it will be seen that they have uniformly been observed to do during the period of vaccination.

"And 10thly. That various authors have attempted to prove, by their observations and experiments, that small-pox virus may undergo such a deterioration in its qualities, from heat, dilution, age, &c. as to render infection with it no security against small-pox, either natural or artificial, even though it may have been sufficient to produce febrile action, and a varioloid eruption so like that of *true* inoculated small-pox, as not to be distinguishable from them by the appearances which present themselves."

[To be continued.]

### DIVISION II.

### FOREIGN.

Art. III.—*De l'Hypochondrie et du Suicide, &c. &c.* Par J. P. Falret, Docteur en Médecine de la Faculté de Paris, &c.—pp. 512. Paris, 1822.

(Article on *Suicide*.)

Having, in a former paper, given an account of M. Falret's Essay on Hypochondriasis, we now come to Suicide considered as a disease,—the "delire suicide" of our author; and our readers will, we trust, still bear in mind, both that we have reversed the order of the French pathologist, and our motives for so doing.

He who pretends to look on death without fear—lies: such are the words of Jean Jaques Rousseau, and, regarding it as a general (perhaps we might say, an universal,) maxim, its truth is indisputable. The unwearied attention with which the man of sickly constitution husbands his weakness, and the care with which even the most wretched, wasting perhaps under incurable disease, struggles by every ingenious shift to prolong his existence, although he thereby prolongs his misery, are sufficient comments on a text, the truth of which every man's observation must verify. If then this be true, how is it that any one—much more, how is it that so many, rush on self-destruction? This question is answered by M. Falret in the following manner: "But if life presented to man but a succession of sufferings, unmingled with pleasure, can it be doubted that it would be hateful to him? It is, then, happiness, or the hopes of acquiring it, which makes him dread the loss of life. When he experiences only painful sensations,—when his imagination spreads a gloomy veil over the future,—man, deaf to the voice of reason, which would paint a brighter prospect, deaf to justice and humanity, disregards every duty; the longing after happiness overpowers him; and this moral or physical grief, which preys upon him, directs the arm of fury which terminates his existence. Would it not be true to say, in general, that suicide is the delirium of self-love?" We have thus early introduced the author in his own person, because upon this position of suicide being a delirium, or mental derangement, the whole fabric of his essay rests. In order, however, to render his opinion more plausible, he hastens to limit the meaning of the term suicide, which does not extend to maniacs who happen to kill themselves undesignedly in fits of frenzy; nor to those who choose to do so from love of their country, —which last he seems to consider a very meritorious piece of patriotism. Rather an equivocal compliment, by the by, to these same patriots, as well as his classing them next in order to the maniacs. Neither, indeed, are his examples of heroic suicide very fortunate; for two out of the five individuals mentioned were destroyed by other means: Codrus was killed by the enemy, and Regulus was put to death by the Carthagenians. By what accident, then, does M. Falret step forward to save the reputation of these great men from a charge which never could be brought against them, by any one acquainted with the manner of their death?

The plan of the work is as follows:—The causes of suicide are first considered; and then the symptoms which mark this "species of mental alienation," their progress, and the morbid changes of structure they produce, illustrated by dissections. The different means, whether physical, intellectual, or moral, which may be opposed to the progress of the malady, next come under discussion; and, lastly, some detailed histories of suicides, in support of the general doctrines, close the essay. These we shall take in succession, with the exception of the last section, from which we shall select, as we go along, any cases which may serve the purpose of elucidation; likewise craving permission to cull from other sources such additional instances as may seem apposite to the discussion.

*Causes.*—The first among the predisposing causes to suicide is hereditary tendency, the reality and extensive operation of which is proved by the authority of various respectable writers; the most unequivocal instance, however, is one which is contained in the concluding part of the work, but which, from its being particularly striking, we shall introduce here. M. R. born of parents, healthy but of taciturn disposition, had five sons and a daughter. The eldest son entered into business at Montaubon; he married, and had several children. His family and friends, knowing that he had made many attempts upon his life, watched him, but at length he threw himself one day from the third floor of a house into the court, and was killed on the spot. This man's affairs were in a prosperous state; he was of good morals, and his melancholic temperament alone appeared the cause of his death. The second son, of a bilio-sanguineous constitution, and of taciturn disposition, was likewise in business: he married, and experienced domestic chagrin; he was said to be jealous. He lost part of his fortune at play, and strangled himself in his own house, at the age of thirty-five years. The third, a man of bilious temperament, precipitated himself from a window, but escaped with little injury: he said he had been trying to fly. The fourth attempted to fire a pistol into his throat, but was prevented. The fifth seems to have lived quietly, and attended to his business; and the sister showed no signs of the disease; but a cousin-germain destroyed himself. From these instances alone, were there no others on record, it would be sufficiently proved that the tendency to suicide may occasionally prevail among several members of a family. To the various examples given by our author we may add one which occurred a few years ago in the north of England, and which, we doubt not, is in the memory of many of our readers, having been published in the newspapers at the time. Two brothers, who were both farmers in flourishing circumstances, and of unimpeached character, went out together early one morning; and, not having returned at the usual time, they were sought for, and found lying near each other in a field, with their throats cut, and their watches on the ground at a little distance, apparently for the purpose of enabling both to commit the act of self-destruction at the same moment. It was afterwards ascertained that the razors, which were found beside them, had been sent to be sharpened but a few days before; and so little appearance of derangement had been evinced, that, notwithstanding every wish to evade the law, the jury were constrained to bring in a verdict of *felo de se*, and they were buried in the highway.

The melancholic temperament is asserted by M. Falret, and no doubt with justice, to be a powerful predisposing cause to suicide; in illustration of which he thinks no other example necessary than that of Chatterton, whose history, he tells us, "is little known, and which however merits to be so on many accounts." Having this authority for its importance, we shall relate the leading incidents of his life in few words, taken from CAMPBELL's "Specimens of the British Poets." Chatterton was born in 1752, and was educated at a charity-school in Burton; from his tenth year upwards he showed a great passion for reading; he was at first very religious, and then became an infidel. He was bound apprentice to an attorney, but, disliking the employment, he left it, and came to London in his eighteenth year, where he engaged in political writings against the government. He was reduced to great penury, and his voluntary death was preceded by great suffering from hunger. On the 25th of August, 1770, he was found dead in his bed, having destroyed himself by poison, at the age of seventeen years and nine months. He had written poems affecting an antiquated style, and given them as specimens of the Gothic muse: these pieces brought him into immediate notice; and he seems to have been of a sanguine and romantic turn of mind, which led him to form bright expectations of future fame and fortune, which were but too soon converted into bitterness and want. "During the few months of his existence in London," says his elegant biographer, "his letters to his mother and sister, which were always accompanied with presents, *expressed the most joyous anticipations.* But suddenly all the flush of his gay hopes and busy projects terminated in despair." In revising the history of this extraordinary boy, we can see nothing but the effect of disappointment on a sanguine, and we fear unprincipled mind. However instructive, therefore, it may be as an example of early suicide resulting from these causes, we cannot but feel astonished that M. Falret should choose it as a specimen of *melancholic* temperament leading to self-destruction; such view of it being by no means unequivocal.

Having discussed the subject of temperaments in this, rather cursory, manner, our author proceeds to inquire into the relation borne by different periods of life to this disease. It would appear that no age is free from it; instances being related of suicide at seven or eight years old, and the father of Barthez, the physician, starved himself to death at the age of ninety, out of grief for the loss of his second wife,—an instance of conjugal love which certainly merits to be recorded. It is, however, when youth gives place to manhood that the disposition to suicide is greatest.

Sex is next considered as a cause; and it appears, both from the observations of Falret and his master Esquirol, that suicide is about three times more frequent in men than women; corresponding to what has been before stated with regard to hypochondriasis.

From sex our author naturally passes to education, in which the reading of romances, the immoderate cultivation of music, and frequenting the theatres, are condemned. The first probably operates by weakening the mind, and giving ideas of sentimental refinement which are not realized when the young visionary enters into this plain matter-of-fact world of ours. Music likewise elevates the minds of some to morbid sensibility. M. Roubaut mentions, in his Medico-Philosophical Researches on Melancholy, that he knew a lady who experienced, three several times, at distant periods from each other, a strong inclination to self-destruction from hearing some airs in an opera. That the representation of tragedies in which suicide is held up to admiration may operate very powerfully, must be acknowledged, when we remember that, in this country, "what Cato did and Addison approved must be right," was the apology left in writing by an unhappy man who destroyed himself.

Climate has been very generally supposed to exert great influence on the tendency to suicide, but certainly without sufficient grounds, as M. Falret shows by two instances in point. Suicide was very prevalent among the ancient Romans, and is not so among the moderns. Suicide is still prevalent in England, while among the Dutch it is comparatively rare, although the climate is very analogous. The number of suicides has been doubled at Copenhagen within the last twenty years, and has of late rapidly increased in France. Although it would appear from these remarks that climate has but little influence, yet it is probable that the seasons are not altogether without effect. MM. Fodéré and Duglas observed that suicides were more frequent at Marseilles when the thermometer was low; and Cheyne states the same thing with regard to autumn and easterly winds. Indeed, "gloomy November" has become almost proverbial for the effect it produces on the spirits.

Among the direct occasional causes, none probably are of more general application than the passions. Of these, the first in power, and most general in its fatal influence, is love. "Let an obstacle be opposed to the desires of a passionate lover," says M. Falret, "or let distrust affect him, and his fury bursts forth; or else, concentrating his griefs, he plunges into solitude to meditate at leisure on the fatal image that never forsakes him. If this condition remains for any considerable time,—if tranquillity be not quickly restored,—seeing nothing but a futurity of misfortune, he seeks in death that repose which he cannot taste under the influence of an exclusive passion." The truth of these remarks is too obvious to require comment, although it is worthy of notice that this destructive tendency of love is by no means confined to the ardour of youth and "hey-day of the blood," but that this passion seems frequently to be most violent after the season of life has passed away at which an effervescence of the affections is naturally to be looked for. "Les vieux fous sont plus fous que les jeunes," says Rochefaucault; and an instance of this, that provokes a smile notwithstanding the catastrophe, is related by M. Falret. In the south of France, a virgin of fifty very lately hung herself at the door of her lover, because he would not consent to unite his fate with hers.

Conjugal love is next considered as a cause of suicide, and the paragraph, as our readers may anticipate, is sufficiently short. However, there are instances of this kind on record; and among these the author introduces the story of Paulina, who wished to die with her venerable husband Seneca. Paulina did magnanimously insist upon her veins being opened,—but it rather mars the beauty of the episode, that she likewise suffered them to be closed again.

Jealousy, ambition, humbled pride, and sense of dishonour, anger, gambling, shame, and remorse, are severally considered, and examples of their influence quoted. Among these, shame is regarded as a cause of suicide chiefly among soldiers whose military exploits prove unfortunate: indeed, these are the only cases alluded to. In man, the loss of honour is dependent on the suspicion of personal cowardice; and so far, we doubt not, M. Falret is correct: but, in woman, it is the loss of chastity which constitutes dishonour. How is it that the author has totally omitted this? It is true he cites the examples of Sophronia and Lucrecia, but these are not to the purpose: the former died to preserve her virtue, and the latter because her person had been violated by the ruffian Sextus. He likewise alludes to the miseries of prostitution; but neither is this the point:—it is the shame following seduction of which we speak. In this country we are satisfied, not only that it is a kind of shame which leads to suicide, but that it is the kind which produces among females more cases of self-destruction than all the other causes put together. Yet we do not question the accuracy of our author, but rather argue from his silence that this cause does not operate with equal force in France. Here, when pregnancy takes place, the means of concealment are extremely difficult, (for we have no "Hospice des Enfans Trouvès,") and the disgrace is irreparable; and hence it is that seduction, under such circumstances, leads very frequently to suicide or to prostitution; and, generally speaking, there is no medium. In France, the opportunities of concealment are greater, and the shame attending discovery less; and hence the loss of chastity is not attended with the same damning consequences as with us.

> "C'est l'opinion du monde qui fait tout l'offence,
> Et ce n'est pas pescher que pescher en silence."

Under the head of "Vague des Passions," we have the story of Renè, in the words of Chateaubriand, which occupies not less than seven pages, and which, being already condensed, does not admit of further pruning: on this account, we must crave permission to pass on to "Domestic Chagrin," a very fertile subject. "Under this name are comprehended all the inquietudes, all the cares, all the dissensions in a family; and they prove very frequent causes of mental alienation and suicide. The nature of these causes, the constancy of their operation, account sufficiently for their great energy. Family disputes, increasing in an infinite degree the cares of housekeeping, contribute to the increase of suicides in France." (p. 54.)

Domestic quarrels naturally lead us to "Reverse of Fortune," the influence of which is shown by a number of classical illustrations; of which, by the by, the author is rather too fond,—as, *cæteris paribus*, we cannot see why Hannibal swallowing poison at the court of Prussia, some centuries ago, is at all more impressive than a Frenchman cutting his throat in 1822: but this is matter of taste. Change of fortune from poverty to affluence is asserted to be a more frequent source of suicide than the reverse.

The operation of indirect occasional causes have, in M. Falret's judgment, been much exaggerated.

"The frequency and energy of these causes have been singularly over-rated: their proportion is scarcely any thing in comparison with the causes which are direct or cerebral. A great influence in the production of madness, and suicide in particular, has been attributed, for example, to the abuse of spirituous liquors; and I myself long believed in this, guided by the general calculations got up (dressés) in France and England; but deeper researches, and the attentive examination of the observations which I had collected, or of those which have been published by other physicians, compel me to think that greatly too much importance has been assigned to this cause. Almost always I have been able to trace a moral affection as the true cause of these mental diseases. It must be obvious that I cannot subject to analysis the facts which authors have cited as favourable to their opinion; but I invite all those who do not adopt my view to take this task upon themselves. The inquiry is, as will be perceived, of great interest in the discovery of the primitive seat.

"Thunberg, in his Travels to Japan, informs us that the Indians, who use opium in excess, are sometimes thrown into such a state of frenzy, that they beat themselves, and mutually endeavour to kill each other; so that very severe punishments are inflicted for this abuse of opium. M. Olivier has observed, that this narcotic very speedily brutalizes man, reduces him to extreme emaciation, and sometimes finishes by drying up all the sources of life. A similar remark has been made by Ananian. We do not deny that the abuse of opium, as well as of spirituous liquors, may develop a predisposition to indifference to life, by exciting the nervous system too powerfully; but, in order that the reader may not be led into error, we must remark, that, independently of the action of the narcotic, there exist almost always exciting cerebral causes, which travellers have not taken into the account." (pages 60—62.)

Bodily pain is a much less frequent source of self-destruction than moral anxiety, although Rousseau was not quite correct when he said "on ne se tue point pour les douleurs de la goutte." Some other indirect causes are alluded to, as pellagra, indigestion, and loss of beauty in women. The author then continues—"This is all that I can say at present with regard to the indirect occasional causes. I hope I have said enough to show the little influence of certain substances, which, introduced into the digestive organs, exert their influence in a secondary manner upon the intellect; and I hope I have demonstrated anew how great the energy is of morals and intellectual causes,—that is to say, of the functions of the brain, on the production of suicide; appreciating at its just value the influence of physical pain.

Among the general causes of suicide, its form of government in different countries seems to exert a considerable agency. The tendency of a despotic government is to repress the development of the passions, except in those dreadful periods of revolutionary struggle in which tyranny is overthrown, or a free people enslaved. These, however, cannot be regarded as the effects of the restricted state of feelings to which we have alluded, but of the effort to escape from it. Tyranny and oppression, however, operate in another way, more calculated to give rise to suicide than the restraint they impose is to prevent it: we mean, by reducing the actual value of life, by the frequency of capital punishments inflicted for slight offences,—as among the Japanese for gambling; and still more by habituating the mind to images of death. May not the public executions, so often witnessed in this country, have a pernicious effect upon the minds of the people? M. Falret is of opinion that a military spirit, by inspiring contempt for life, may become an occasional cause of self-destruction, and cites the frequency of suicide among the Romans in illustration of his position. The accuracy of this appears to us extremely questionable; for, if we turn to the history of Rome, we shall see that it was not the soldier, so often as the philosophic citizen, who embraced a voluntary death: or, if we meet with men of military genius occasionally doing so, still even here we shall find them to have been devoted to study and reflection,—such, for example, were Cato and Brutus; and our author acknowledges that scarcely any examples of suicide were met with in the calamitous retreat of the French from Moscow; while the same general remark of the unfrequency of this occurrence applies with equal truth to our own armies, whether in prosperous or adverse circumstances.

Religious fanaticism has at all times, and under every diversity of belief, been a very fertile source of suicide. Designing men too often work upon the imaginations of simple and credulous people, many of whom are thus led to regard themselves as guilty of imaginary crimes, and set aside as "vessels of wrath." "Many theatric preachers among the Methodists, says Dr. Darwin, successfully inspire this terror, and live comfortably upon the folly of their hearers. In this kind of madness the poor patients frequently commit suicide, although they believe they run headlong into the hell which they dread! —such is the power of oratory, and such the debility of the human understanding!" From the time of Henry the Eighth, this country has been much divided by religious sects, and this is to be regarded as one of the chief causes of the frequency of suicide, which first became remarkable about the middle of the sixteenth century. More recently, it is probable that the metaphysical speculations of Hume, and the sceptical doctrines to which they gave rise, may have contributed in a considerable degree to the same effect.

In France, the crime of self-destruction was particularly prevalent both about the time of the revolution and since the overthrow of the Napoleon dynasty; and, although our author does not allude to it, we have no doubt but that the wild enthusiasm of ROUSSEAU, and the profligate sophistry of VOLTAIRE, paved the way for that increase of suicides among their countrymen which they do not themselves affect to deny.

It has recently been made a question between MM. ESQUIROL and FALRET on the one hand, and Dr. BURROWS on the other, whether suicide be more frequent in Paris or London. In this discussion, we shall confine ourselves to a few remarks on what appears in the work before us, endeavouring to divest ourselves of those national feelings by which it is quite evident M. Falret was influenced. Dr. Burrows, he says, "*n'a pas craint d'assurer que la folie et le suicide sont moins multiplies dans sa patrie que dans notre belle France;*" while this daring assertion is said to have been made in an "Inquiry relative *of to* Insanity." One would not suppose a mere matter of fact likely to give rise to much discussion, and we are therefore somewhat suspicious of reasoning which is preceded by declamation. We request any impartial reader to observe the following facts.— M. Falret gives official tables, by which it appears that in the department of the Seine, in 1817, the number of suicides amounted to 351; and, in 1818, to 330; while, by a similar document, it appears that, during the first six months of 1819, the number had arrived at 199. M. Falret erroneously asserts that Dr. Burrows had selected 1813, and thus protests against his own delinquency, "ce médecin a d'ailleurs commis une erreur très grave en comparant le nombre des suicides executés dans le cours d'une année determinée a son choix, en 1813;" whereas 1817 was the year selected, because it enabled him to compare Paris and London with Berlin, M. KAMPFER having published tables of the suicides in Germany for that year. Yet we are astonished that M. Falret should object to 1813, which he (from inadvertency, we must suppose,) has asserted to be the year chosen by his opponent, as there were fewer suicides in Paris then than during any season since that time. We shall take 1817, which may probably be about the medium, as there were more in 1819, and fewer in 1813. In 1817, then, the French acknowledge 300 suicides in Paris: in London, the number of suicides entered in the Bills of Mortality amount to forty; 100 persons were drowned, and of these Dr. Burrows calculates that "forty may have met a voluntary death;" 120 are registered as having died insane, and Dr. Burrows sets the whole of these down as suicides, and thus calculates the total number at 200. To this method of calculation M. Falret objects, and asks, "on what ground does he fix the number (of those registered as insane who committed suicide,) at 120?" Why, simply because that, being the whole number, he was quite sure that he did not make the calculation too favourable for his own views. But, to satisfy M. Falret, we shall give him likewise all the drowned, and then the calculation with regard to London will stand thus:—Registered suicides, 40; drowned, 100; died insane, 120; regarding them all as having destroyed themselves, 260, still, even this makes the number of suicides during 1817 forty more in Paris than in London. But the calculation in London is evidently too high; for, in a commercial city, where there is such constant traffic on the river, it is quite certain that a large proportion of the hundred who were drowned must have met their death through accident; and, as mad people die as well as others, it is very improbable that the whole of the lunatics met their death by self-destruction. Those interested in the subject we refer to the original works of Esquirol and Burrows, and to the paper by the latter in our Number for December 1822. With regard to M. Falret, we are quite satisfied that he has not read the "Inquiry" of Dr. Burrows, which he attempts to refute, but that the whole is taken from the article on Suicide by M. Esquirol, in the Dictionnaire des Sciences Medicales. The circumstance of Dr. Burrows' book being there likewise entitled an "Inquiry relative *of to* Insanity," is quite convincing to us: our author has copied his master even in his errors. We are of opinion, from the result of this inquiry, that of late suicides have been considerably more numerous in Paris than London; and, consequently, (keeping in view the difference in the population,) that the proportion is very much greater. The following is the estimate of Dr. Burrows for 1817:

| | *Population.* | | *Suicides.* | | *Proportion.* |
|---|---|---|---|---|---|
| Paris,------ | 700,000 | — | 300 | — | 0.42 in 1000. |
| London, -- | 1,000,000 | — | 200 | — | 0.2 in 1000. |

The subject of Ennui occupies many pages, but we shall be very brief, lest we give to our readers a more perfect idea of this state of mind than we desire. No one can judge of another's enjoyments, even from the most promising external circumstances; and we probably call many an individual happy who in reality is sick at heart. Our author is evidently partial to the philosopher of Geneva, and we are rather astonished that he has not here impressed into his service one so well qualified to speak on this subject. Amid the sophistry of Rousseau there are many sentiments of beauty and truth, which show him to have been well acquainted with the human mind, and such is the following: "Le bonheur n'a point d'enseigne extérieure —pour en juger il faudrait lire dans le cœur de l'homme heureux.

*Symptoms and Progress.*—The forms of suicide are infinitely varied, but, according to M. Falret, they may be divided into two general varieties, extremely different from each other. The importance of these remarks induce us to give them in the author's own words.

"Different suicides present infinite varieties, which are better illustrated by an examination of the causes than by description; but, generally speaking, the propensity to suicide, like melancholy, of which it is often but the last stage, is capable, in my opinion, of assuming two principal but opposite forms: one is characterized by the most profound sorrow, a state of dejection and fear, and a particular passion for solitude; the other by great excitement, both physical and moral. This last kind of suicide takes place suddenly, as the event of the storm of some passion. The symptoms which characterize it are as variable as the passions in which they originate. The attention which I have given to their study,—the care with which I have investigated their mode of action especially, ought to spare the necessity of extended details. In these cases, the suicide is generally executed as soon as it is resolved, and the physician is only called to witness its terrible effects. In other circumstances the progress of the delirium is more gradual; the observer may perceive its character, and arrest its course. It is remarked, in general, that the countenance of the patient possesses an extreme degree of mobility, and even appears somewhat convulsed. Parkman relates, that Lord M. having requested the painter Stuart, a distinguished artist in London, to make a portrait of his brother, Captain C. P., the painter took advantage of his intimacy with the captain, to delineate the animation and mobility of his features. Lord M. seeing the portrait, remarked, 'This is not the portrait of my brother; *it is like a madman.*' The painter requested another sitting: his lordship saw the picture again, and exclaimed, '*My brother appears more mad than before.*' Three weeks after, the captain blew out his brains. The face is generally red, the eyes injected, with throbbing of the temporal and carotid arteries. The head ache, which almost always troubles these patients, varies in degree and in its situation, but the forehead is generally the seat of the most severe pain. Watchfulness is likewise almost constant, and sometimes precedes every other symptom. The sensibility is apt to experience great changes; sometimes it is almost entirely blunted, and at others acquires an increase of vivacity. It is not uncommon for the patient to experience either an icy coldness or a burning heat over the whole body, or in certain parts of it; and even a kind of commotion from the feet to the head. Some say they have experienced inexpressible anxiety for some time previous to attempting their life. They felt their heads become confused, and sought to destroy themselves, as well to be delivered from their actual evils, as overpowered by the unhappy ideas which tormented them. Others, on the contrary, feel a kind of joy, and seek death as a shelter against the tempest.

"Independently of the symptoms referrible to the head, we pretty frequently observe lesions more or less marked, but in general very slight, in the organs of the abdomen and chest. Some signs of indigestion are remarked; the hypochondriac regions are the seat of unusual heat; sometimes they are hard, distended, and painful. This last symptom is far from being constant, as has been so often stated; and we shall examine elsewhere if a great error has not been committed, in regarding the liver or the spleen as the primary source of the cerebral disorder. The chest is sometimes in a state of remarkable constriction; but, in general, during the continuance of the paroxysms, the organs contained in it appear gifted with new energy: the beating of the heart is stronger, more frequent, and tumultuous; and the respiration participates in the activity of the sanguiferous system.

"The progress of suicide with excitation is much more rapid than that of the disease with depression and sadness. The prognosis is more favourable: for, if the inclination to suicide does not degenerate into mania, it usually ceases with the cause which has produced it. It is likewise true that it easily assumes the intermittent type,—that it is liable to be reproduced by the slightest causes, and, after many returns, to become habitual." (p. 111—115.)

The questions, whether the act of suicide be ever really committed with coolness? and whether it be a proof of courage? are next discussed; both of which the author is disposed to answer in the negative. The wavering and doubtful condition of a timid mind is well described, and fully illustrated. It seems probable that persons of this disposition are so much affected with contemplating death, that they prefer embracing the evil at once, rather than sustain the prolonged anticipation.—Rousseau, as usual, is brought in, and an account of his death given, from the writings of Madame DE STAEL—almost as great a sophist as himself, and one who, when a young woman, favoured the world with some effusions in favour of suicide. Rousseau was impatient under bodily pain and mental vexation, and his apology for desiring death is thus concluded: "Puisque mon corps n'est plus pour moi qu'un embarras, un obstacle à mon repos cherchons donc à m'en degager le plus tôt que je pourrai." We crave our readers' indulgence for one more quotation from Rousseau, not given by Falret, but taken from "La Nouvelle Helouise," the author of which not being so great a favourite with us as he seems to be of his countryman, we promise not to introduce him again. "Le suicide est une mort furtive et honteuse. C'est un vol fait au genre humain. Avant de la quitter, rends lui ce qu'il a fait pour toi. * * * Philosophe d'un jour! ignore tu que tu ne saurois faire un pas sur la terre sans trouver quelque devoir à remplir, et que tout homme est utile à l'humanité par cela seul qu'il existe?" Let these two paragraphs be contrasted, and the weakness and inconsistency of their author will be obvious.

The author proceeds to show that the violent death of maniacs ought not to be regarded as suicide, unless it depend upon the will; and next treats of the connexion between hypochondriasis and suicide. The former of these positions requires no illustration, and the latter circumstance has been already considered in our preceding Number.

Among the phenomena connected with this subject, there is probably none more remarkable than the disposition frequently evinced to involve those most dear in the same destruction. A remarkable instance is that of Richard Smith and his wife, who first killed their child, and then hung themselves. Various other instances are given by our author; one of them may suffice. "In the month of April, 1816, Madame ——, aged thirty-six years, and remarkable for the vivacity of her disposition, the regularity of her features, and the expression (at once soft and imposing) of her countenance, dragged three of her children towards a well; a boy of four years old, and two girls,—one, nine years of age, the other, five. The two youngest were first thrown in; the eldest struggled a long time in her arms, but this unfortunate mother, using more force, threw her in next, and ended by precipitating herself." They all perished; and it was discovered, some hours after, that the mother had endeavoured to procure her infant, whom she had sent out to nurse, doubtless with a view of destroying it; and that she had sent a poisoned cake to her son at school.

That the exciting causes of suicide may prevail under such circumstances as to give it the appearance of prevailing epidemically, is sufficiently proved by the records of medicine. Allusion to this circumstance is made by Plutarch, Montaigne, Primrose, Sydenham, &c. In 1806, sixty suicides were committed at Rouen, during the months of June and July, which had been extremely hot and moist; much commercial distress had occurred at the same time. In July and August of the same year, three hundred suicides were committed at Copenhagen; the same causes likewise prevailing. In 1793, thirteen hundred suicides took place at Versailles!

The *prognosis* is thus spoken of:

"The prognosis, in the delirium of suicide, is always very unfavourable, as this disposition results from perversions of the functions connected with the affections, and from the last stage of melancholy. It will be less so, however, if the suicide be not complicated,—if it be recent,—if it depend on moral causes which are slight, and does not arise from vicious education. In general, suicide is not obstinate when it is the first symptom of madness or melancholy; particularly when, at this time, the patients wish to starve themselves to death. It is often sufficient, under these circumstances, to excite the action of the stomach or intestines, by an emetic or appropriate purgative. It sometimes happens that deranged persons, who wish to destroy themselves, but fail in the attempt, become radically cured, or at least are diverted from their unhappy tendency for a longer or shorter time. This fortunate issue is most frequently remarked when the disease is brought on suddenly by some violent passion. (p. 172-3.)

*Changes of Structure, and Seat of the Disease.*—The examination of the bodies of those who have committed suicide, has received considerable attention from our author; but, as they appear to us to prove nothing, we shall not dwell upon them. Heister found the pancreas diseased. Esquirol, Desgenettes, and M. Falret, have seen the colon run obliquely, or even in a perpendicular direction. Professor Osiander has observed the intestines to be affected with chronic inflammation. Corvisart has remarked organic disease of the heart. Gall asserts that the cranium is thick and dense; M. Freteau has remarked stagnation of blood in the head; and, lastly, others have found no morbid appearance any where. From all this we conclude, that the diseased alteration of structure which gives rise to suicide (if there indeed be any such,) is still imperfectly unknown. Such being the case, M. Falret adopts the method of determining the seat of disease which we noticed with regard to hypochondriasis, in the preceding part of this review: that is, he endeavours to ascertain the occult phenomena of the disease, by reasoning on the symptoms which are apparent. In order to do this with advantage, he regards it as essential to take into consideration the following circumstances:—1st. The predispositions, natural or acquired; 2d, the occasional causes of the perceptible derangements, their nature, and mode of action; 3d, the order and succession of the symptoms, and the phenomena of the sympathies; 4th, the termination of these maladies, and the nature and mode of action of the various curative means adopted; last in order, and least in importance (according to M. Falret), come examinations after death. Reasoning in the manner pointed out under these heads, the author proceeds to examine into the consequences which are legitimately to be deduced from the histories of cases of suicide. To do this, he gives ten cases from the works of Fodéré* and Leroy,† which they have related to show that the primary cause of the mental alienation leading to suicide, is to be sought in the abdomen. The method of M. Falret will best appear from an example. "The subject of the first

† *Bulletin des Sciences Médicales de la Societé d'Emulation de Paris,* Juillet 1808.

observation is a man about sixty, who, *having lost a sum of money which he had lent to one of his friends, became by degrees melancholy;* he lost his appetite, sought after solitude, and ended by hanging himself," &c. We have placed in italics the same words which are so marked in the original, which method is adopted by the author as a sort of running commentary, intended to point out the existence of intellectual or moral causes; that is to say, the existence of symptoms referrible to the head. Italics, as might be expected, are to be found in every case, and we think M. Falret succeeds in his illustrations with regard to all, except one, in which he himself remarks that the cause of the attack is not assigned. After these follow twenty cases from other sources, to show the mental origin of the disease, (it would be easy to multiply the number *ad infinitum;*) and, what is more to the point, a discussion tending to illustrate the fact, that primary affections of the brain, from other and more apparent causes, as external violence, frequently produce the same alterations of structure in the abdominal viscera as his opponents have described in suicide; and, consequently, affording our author an additional argument in favour of their secondary origin.

*Curative Means.*—In pity to our readers, we pass over various proofs of the brain being the physical seat of mind, and an agent in the operations of thought, and proceed to the treatment; which, considering what has been said in the preceding part of this article, need not detain us long. The general indications differ in nothing from those laid down with respect to hypochondriasis. Manual labour, the chase, travelling, and similar methods of distracting the mind from indulging in melancholy trains of thought, are to be enjoined. Purgatives are a sort of universal prescription, and are not omitted here. Emetics are said to be of signal utility in preventing a relapse; and, if the medicine does not excite vomiting in the usual dose, we are advised to give a small quantity of opium before repeating it, as suggested by M. AMARD, of Lyons. Bleeding, blisters, setons, and similar remedies, are spoken of as occasionally useful; and the author, somewhat inconsistently, recommends their application to the hypochondrium. DARWIN mentions an instance of a gentleman who had an issue on the head, which dissatisfied him very much, and he suffered it to heal: when it had done so, he cut his throat.

These methods are applicable in the interval between paroxysms of this species of insanity. The conduct of an attendant at the moment, or while a patient is brooding over the means of destruction, requires much discrimination and presence of mind. As an invariable principle, they are not to be left alone nor unguarded: recent calamitous examples in our own country might be quoted in illustration. With men of honourable mind, it is frequently possible to turn these feelings to account, exacting from them a promise not to attempt suicide. This remark applies more especially to those affected with spleen: where the derangement is of a more violent kind, such restraints would be but feeble. The author of the "Zoonomia" states, that a gentleman, apprehensive that his friend meditated self-destruction, obtained from him a promise that he would not do so before four o'clock next day. He fulfilled his promise, but blew out his brains as soon as the hour was expired.

It becomes extremely difficult to gain the confidence of the patient, and yet withhold from him those instruments by which his purpose may be effected. No rules can well be laid down: we must be guided by our knowledge of the patient, and the circumstances of the moment. The late King asked for razors:—to hesitate, was to suggest the idea of suicide, which probably had not occurred to him; to give them, run the risk of the purpose being effected, if it had been conceived. Dr. Willis sent for the razors, and mean time endeavoured to engage his Majesty's attention on some other subject; and, having succeeded in doing so, became convinced that he had no intention of using the razors for any purpose of violence. M. Falret was once placed in similar circumstances. A person, whom he knew had the design of destroying himself, asked him for razors to shave with: trusting to his knowledge of the case, M. Falret complied. "The patient, after having shaved, looked at me, and observing that I remained tranquil, he exclaimed, 'How well you know me!' From this time I had all his confidence."

In speaking of the means of restraint sometimes necessary in the cases of refractory patients, M. Falret insists much on the adoption of those which are the most gentle. The revolving machine is first mentioned, with some approbation, and the cases specified in which it is most likely to be of use; but, turning to the next page, we find that in the interval he had been to Zurich, where a trial of the machine on his own person had made him alter his opinion. Instead of putting his pen through the preceding remarks, he undoes them by the following philippic:—"Since this was written, I have had occasion to judge for myself of the effects of the rotatory machine at the Hospital for Lunatics at Zurich. Although every precaution had been taken, I felt a frightful uneasiness: I was in imminent danger of apoplexy. The remembrance of this painful state will make me henceforth resist the seduction of authority, and beget more reserve in the employment of energetic and perturbating means,"* (*moyens energiques et perturbateurs.*)

* We have reason to believe that this machine has never been employed in this metropolis.—EDITORS. [illegible]

Under the head of Preservative Treatment, it becomes a question, whether the enactment of laws against suicide have any beneficial influence in preventing its occurrence. The author remarks upon the views taken by the governments of different nations on this subject. In Greece, suicide was looked upon as a crime, and the body of the deceased treated with dishonour: in Rome, it was regarded as an act of heroism and philosophy. The legislature of every nation of modern Europe has discountenanced the crime; and, while M. Falret admits that this may occasionally arrest the arm of suicide, yet he protests against such laws, as being, in the great majority of cases, "unjust, useless, and even dangerous." The importance of the subject will, we trust, induce our readers to grant us their attention a little longer, while we inquire into the grounds on which this opinion rests.

"Let us see if there are not more solid reasons in favour of our opinion. The ancient laws against suicide were cruel, and unjust towards the family of the deceased, whom they despoiled for a fault of another. The son, in deploring the loss of his father, had also to lament the loss of property. They punished the widow for the loss of her husband; and sometimes their cruelty even went so far as to strip her of her own private fortune.

"It was not enough, the unjust confiscation of goods; the ignominy of the hurdle disgraced an innocent family. Astonishing blindness! the legislature punished with more rigour an unhappy man who committed suicide, than a ruffian who had imbued his hands in innocent blood. The family of this monster was not the object of the vengeance of the laws; but the family of the insane was visited with all their severity. * * The violent death of a beloved parent,—confiscation of his property,—and, above all, the infamy attached to his name and his posterity;—can a concurrence of circumstances more calculated to excite suicide be imagined?

"At present it is possible, to a certain extent, to conceal from the children that there has been a suicide in the family; but, if you give it more publicity by the execution of a rigorous law, the children will inevitably come to know it, and this frightful intelligence cannot but augment their own lamentable predisposition. * * What, is it allowed that suicide is the most hereditary form of insanity, and yet we invoke all the severity of the laws to punish it! Would we, then, that society should hasten to mark its victim in the mother's womb? * * *

"The laws have no restraint in arresting crime more powerful than the fear of death. This check does not exist for him whom a firm and disordered will prompts to suicide. What law but would prove insufficient, when he has triumphed over the instinct of self-preservation? Where seek the means to restrain the arm of one who has already broken the strongest tie that binds him to life?" (p. 270, &c.)

Now, with regard to the two first paragraphs, we would observe, that they may perhaps prove that bad laws have been enacted, and that such have produced no good effect: they certainly prove nothing further. The third objection, that we may at present conceal from the children the ignominy of the parent, but that this could not be done if the crime was punished by law, is weak in the extreme: the children, being the most interested, and the most with their parents, are generally the first to discover the cause of death. The possibility of concealing it from them does not exist once in an hundred times. With regard to the alleged injustice of punishing a crime supposed to be hereditary, and to begin "even in the mother's womb," M. Falret will please to remember that he is himself the great advocate for moral means; and what moral check is more powerful to men who have any good feelings left, than the certainty of bringing infamy upon their memory, and disgrace upon their relatives? In the last clause quoted (and we request our readers to believe that we have given those which appear to us to support most strongly the opinion of M. Falret,) the author puts the fear of death, as a penalty of the law, upon the same footing as the fear which a person meditating self-destruction may be supposed to entertain; than which nothing can be more incorrect. The one is a *punishment*, unsought for, and having no choice left as to the time or means of its execution: the other a voluntary act, committed for the purpose of escaping from present misery. We can easily conceive that a person shall have such a dread of the gallows, as to make his escape from prison, and afterwards hang himself at home. We remember, not many months ago, reading an account of a watchman in Westminster, who hung himself in his room, but, being fortunately discovered, was taken down; and, having changed his mind, went the same evening to his usual duty. Can any one suppose that, if it had been proposed then to hang him at Newgate, he would have regarded the operation in the same light which he did the voluntary gratification of his own free choice. We, therefore, deny the parallelism between capital punishment and suicide; nor do we admit the fear of death as universally the greatest possible restraint: on the contrary, we are convinced that many who would have courage to destroy themselves, with the prospect of leaving an unspotted reputation, would shrink from it under the certainty of their name being branded with infamy, and their nearest and dearest relatives sharing in the disgrace.

But the question is not, whether the effect of unjust or severe laws, as quoted by the author, be favourable to the purpose for which they are intended; nor whether the operation, even of the mildest punishment, be agreeable to the feelings of surviving friends; but whether laws of any kind have a tendency to prevent the crime of suicide? It is thought by M. Falret that they have not; and, among other reasons for this opinion, it is stated that, in France and England, where the laws have been most severe, the number of suicides has been greatest. But when we consider that in this country the law is almost always evaded, while in France it was repealed above thirty years ago, the argument loses all its force. That an opinion prevails in France, that the great increase of suicides in that country is, in some measure, owing to the disuse of the ancient laws against it, is proved by the fact that a petition for their restoration was presented to the Chamber of Deputies in 1819. Again, we are told that the number of women, in India, who burnt themselves on the funeral piles of their husbands, was increased by the attempts of government to repress this barbarous practice; but this is a case altogether peculiar; it is an instance of the pertinacity with which many eastern nations adhere to ancient customs and rooted prejudices; it shows, not that laws are ineffectual, but that they prefer their own law-givers to ours. At all events, it bears no analogy to the causes of suicide in Europe.

The author compares the enactment of laws against suicide and duelling, as equally mistaken in principle and futile in effect. This may have been the case in France, but certainly does not apply to other countries. The infliction of the sentence of death on Colonel Campbell, and the marked disapprobation of the illustrious individual at the head of the army, have tended much to diminish the frequency of duelling in England; while the same effect was produced in Russia, by the severe enactments of that civilizing savage, the Czar Peter.

In the work before us, it is attempted to prove by reasoning that laws are useless in the case of suicide; yet two facts of a positive kind are related, which are to us stronger than the arguments, which at best are but negative. The young women of Miletus, impatient of the long absence of their lovers in the wars, had recourse to suicide, which prevailed among them to a great extent. The senate, anxious to stop the growing evil, decreed that the body of every girl who destroyed herself should be exposed naked in a public part of the town. The effect was immediate, and the damsels were content to bear the miseries of separation, rather than the violation of modesty. In the tenth year of the French republic, two suicides occurred in one regiment within a month. Bonaparte arrested the contagious example, by publishing in his general orders, "that a soldier ought to know how to conquer grief and the melancholy of the passions; that there is as much true courage in bearing with constancy the pains of the mind, as in remaining firm under the fire of a battery, &c."

M. Falret endeavours to explain away the force of these and similar examples, by arguing that such enactments are but of limited application; and we readily admit, that the exposing the bodies of the soldiers would have had as little effect in preventing suicide among them, as a dissertation on the comparative degrees of fortitude, under affliction and a brisk cannonade, would probably have had upon the young ladies of Miletus.

To these examples, which are quoted by Falret, we may add two others, alluded to by M. Esquirol: the first is, that when the declamations of Agesias had rendered suicide common in Egypt, their evil tendency was effectually counteracted by Ptolemy, who forbad the philosophy of Zeno to be taught, on pain of death. The second occurred in America, where the unfortunate negroes had recourse to suicide, in hopes of returning after death to their native country: an Englishman put a stop to this practice, by causing the hands of those who committed suicide to be cut off, and exposing them to the view of their countrymen.

The nature of the laws to be enacted may be a matter of question, and our author is doubtless correct in supposing that all legislators are not so well acquainted with the genius of the people whom they rule, as the shrewd senators were with the disposition of their countrywomen, or the first consul with the minds of his soldiers. Upon the whole, we are inclined to agree with Frank, Fodéré, and Esquirol, who are all opposed to M. Falret. "Reasoning (says M. Esquirol,) cannot prevail against the authority of experience: certain comminatory laws have put an end to suicide in Egypt, Miletus, and America. Suicide has become more frequent since the laws against it have lost their vigour: for the interest, then, of society, the legislature might in future establish laws against it, not penal, but comminatory. It does not belong to me to suggest these laws; but I think they ought to vary according to the character, the manners, and even the prejudices, of the people, and to be directed against the social causes which contribute to develop the tendency to suicide?"* In Saxony, the bodies have recently been ordered by the king to be given for public dissection:—might not a hint be taken from this suggestion?

The merits and demerits of this essay differing in nothing from those of the preceding, we refer to our review of it for a general character.

* *Dictionnaire des Sciences Medicales.*

