# Erratum

**Published:** 1823-04

**Authors:** 


### ERRATUM.

In our last Number, p. 244, for Court of *Prussia*, read Court of Prussias.

